# Normative data for the 10-min lean test in adults without orthostatic intolerance

**DOI:** 10.3389/fneur.2025.1625216

**Published:** 2025-10-02

**Authors:** Nafi Iftekhar, Amy Wilson, Louis Nguty, Hussain Al-Hilali, Yusur Al-Hilali, Kinshuk Jain, Angela Braka, Thomas Osborne, Manoj Sivan

**Affiliations:** ^1^School of Medicine, University of Leeds, Leeds, United Kingdom; ^2^Leeds Institute of Rheumatic and Musculoskeletal Medicine, Leeds, United Kingdom

**Keywords:** orthostatic intolerance (OI), postural orthostatic tachycardia (PoTS), orthostatic hypotension (OH), lean test, normative data, autonomic dysfunction, dysautonomia, long COVID

## Abstract

**Background:**

Orthostatic intolerance syndromes such as Orthostatic Hypotension (OH) and Postural Orthostatic Tachycardia Syndrome (PoTS) are common symptoms seen in post-infection conditions and other neurological conditions with autonomic dysfunction. The 10-min Lean Test (LT) is an objective clinical test used to assess these symptoms and direct management. There is, however, no robust literature on normative data for this test, particularly from a younger population.

**Aims:**

The aim of this study was to produce a healthy control data set for LT, which can be used for comparison with the patient population with health conditions.

**Methods:**

Individuals recruited into the study had no history or symptoms of orthostatic intolerance; autonomic dysfunction; post-infection conditions (such as long COVID); or other neurological conditions with hemodynamic instability. Participants were primarily recruited from the general population in a metropolitan city. All participants underwent a standardized LT. Lying Blood Pressure (BP) and Heart Rate (HR) after 2 min of lying down supine was recorded, followed by BP and HR recordings at every minute of standing (leaning against a wall) up to 10 min, along with recording subject-reported symptoms at each time point.

**Results:**

A complete dataset was available for 112 individuals (60.7% Female, 39.3% Male). The population was 61.6% Caucasian, 8.0% Asian, 3.6% Black/Caribbean, 9.8% Mixed, and 17.0% Other; the mean age was 35.3 ± 15.1, with a BMI of 24.8 ± 4.0; 30.6% of individuals had a background medical condition, but none of the exclusion criteria. During LT, upon standing, the average change of HR was an increase of 9.89 ± 8.15 bpm. The sustained HR increase (HR increase sustained at two consecutive readings) was an average of 6.23 ± 6.94 bpm. The predominant response with BP was an increase of systolic BP, with the average initial increase being 7.55 ± 10.88 mmHg. None of the participants met the diagnostic criteria for symptomatic OH or PoTS during LT.

**Conclusion:**

For the first time in the current literature, 10-min LT data from a relatively younger population without orthostatic intolerance have been gathered. This normative data will help interpret LT findings in younger patients with orthostatic Intolerance better and be useful in managing dysautonomia in specific conditions.

## Introduction

Orthostatic Intolerance (OI) (1) refers to symptoms arising from the inability to maintain normal blood pressure or heart rate when standing upright, which is then alleviated by reclining or lying down (2). OI can cause symptoms such as myalgia (3), dizziness, syncope (4), fatigue, headache, nausea, and palpitations (5, 6).

Postural orthostatic tachycardia syndrome (PoTS) (7) is a type of orthostatic intolerance characterized by an excessive increase in heart rate (HR) (more than 30 beats per minute increase or actual HR over 120 beats per minute) within the first 10 min of standing. Orthostatic Hypotension (OH) is characterized by a drop in systolic BP of more than 20 mmHg (or drop in diastolic BP by more than 10 mmHg) within the first 3 min of standing. If OH is present, a diagnosis of PoTS cannot be made.

Both PoTS and OH have been noted in literature to be associated with long COVID (LC); a condition characterized by persistent symptoms, lasting greater than 12 weeks, experienced after recovering from an acute COVID-19 infection (8, 9). The common symptoms of LC are post-exertional malaise, fatigue, brain fog, pain and dizziness (10). In the UK, nearly 2 million individuals are reported with LC (11). In an average-sized medical practice in the UK, a general practitioner can anticipate having approximately 65 patients affected with LC (12, 13).

One of the plausible mechanisms in LC is dysautonomia; a term that refers to the dysfunction of the autonomic nervous system (ANS) (14, 15), which regulates involuntary bodily functions such as heart rate, blood pressure, digestion, sweating and temperature. Dysautonomia can have various causes, such as infections, autoimmune diseases, genetic disorders, or trauma (16). PoTS and OH are the common dysautonomia syndromes of the cardiovascular system (17–19).

There are a few proposed theories on pathophysiology of dysautonomia in LC, including direct damage to autonomic nervous system by the virus and indirect damage via autoantibodies (20). There is considerable recent evidence reporting a high prevalence of dysautonomia syndromes in LC (21). The prevalence for PoTS in LC is reported to be approximately 40–50% (22–24) and approximately 40% (24–26) of LC patients have been reported to have OH.

Dysautonomia is also observed in a range of medical conditions such as amyloidosis and HIV to Guillain–Barre Syndrome and paraneoplastic syndromes (27, 28). The OI syndromes of PoTS and OH have been reported in conditions such as Parkinson’s, Multiple Sclerosis and Diabetes mellitus (29, 30).

When assessing patients for OI syndromes, a range of objective tests can be used. The conventional test is the Head Up Tilt (HUT) table test which is carried out in hospital settings. A simpler active stand test (31) or 10-min Lean Test (LT) (13, 32, 33), can be conducted in a clinic or at home setting. Recent studies have validated the use of LT for detecting OI syndromes in LC (1, 34). The need for normative data to interpret the findings of LT has been highlighted in these studies.

The purpose of this study was to produce a normative data set from participants without orthostatic intolerance symptoms, and from a younger population. This will enable us to interpret the LT findings in medical conditions with greater confidence and manage the symptoms more comprehensively.

## Methods

### The LOCOMOTION study

The work reported here was part of LOCOMOTION (LOng COvid Multidisciplinary consortium Optimising Treatments and services across the NHS), a 30-month multi-site case study of 10 LC clinics beginning in 2021, which sought to optimize LC care across the clinics. The study protocol with details of management, governance and patient involvement has been previously published (35). Ethical approval was granted by Yorkshire & The Humber— Bradford Leeds Research Ethics Committee (REC; ref.: 21/YH/0276) and subsequent amendments.

### Participant sampling and consenting

The inclusion criteria for this study were individuals without a formal diagnosis of LC or any condition with dysautonomia or hemodynamic instability. Exclusion criteria for the study were the inability to give informed consent or comply with test instructions, if the clinical team considered the test unsuitable or unsafe (e.g., if the participant could not stand unaided), and any coexisting condition that could interfere with autonomic or hemodynamic function. Verbal and written informed consent to perform the LT were obtained from all participants.

Participants were recruited in a 5-month period from a metropolitan city.

### Sampling

The sampling frame for this study was purposive to match the demographics of LC patients seen in NHS clinics.

### The lean test

The LT was performed according to published instructions (1, 32) and tests were performed at random times during the day. We chose 2–5 min as the protocol of lean test we used for our participants.

All participants first lay quietly for 2–5 min; then one supine reading of HR and BP was obtained, which is used as a participant’s baseline. Subjects then stood slowly, shoulders leaning against a wall and feet positioned 15 cm from the wall. HR and BP measurements were obtained at standing (0 min) and then at one-minute intervals until 10 min of standing had been completed.

### Data collection and management

HR and BP were collected with the Omron M2 Upper Arm Blood Pressure Monitor (HEM-7146-E) (OMRM2+) blood pressure monitor, which has been endorsed by the British Hypertension Society (36).

Data was aggregated through a form created on Jisc Online Surveys with the consent form attached for each submission. The form collected the following information:

- Consent for testing
- Age
- Sex
- Weight
- Height
- Ethnicity
- Current Medical or Surgical History
- Current Medications
- LT Data
- Location of Data Collection

All data were anonymously collected, and information was stored with the University of Leeds secure driver, in line with the ethical guidelines and approval.

### Statistical analysis

Statistical analysis was conducted using GraphPad Prism, including standard statistical tests and significance calculations. Microsoft Excel was utilized for data management and formatting. Results are presented as mean ± standard deviation unless otherwise specified. Kolmogorov–Smirnov tests confirmed normal distribution.

## Results

### Consort diagram

**Figure 1:**
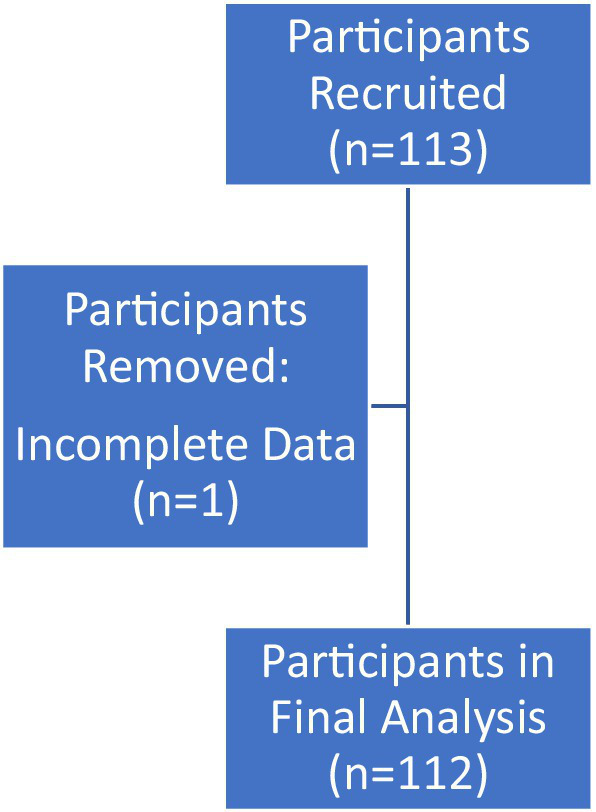
Consort diagram for participant recruitment. *n* = 1 participants were removed for an incomplete dataset.

One hundred thirteen participants were recruited into the study. One participant was excluded as there was incomplete data. No participant had the LT terminated and all participants in the final analysis were able to complete the 10-min standing (Figure 1).

### Participant demographics

Demographic data for the 112 participants (68 female [60.7%]; 44 male [39.3%]; age 35.3 ± 15.1 years) of this cohort are shown in Table 1. Most participants were White (69 [61.6%]), with other ethnicities including Asian (9 [8.0%]), Black/Caribbean (4 [3.6%]), Mixed (11 [9.8%]), and Other (19 [17.0%]). Mean BMI was 24.8 ± 4.0 kg/m^2^ (range 17.8–39.4). Thirty-four participants (30.6%) reported at least one medical condition, with asthma (9 [8.0%]), depression (5 [4.5%]), hypertension (4 [3.7%]), and GORD (3 [2.8%]) being most common. Forty-two participants (37.5%) were taking at least one medication, with metformin (5 [4.5%]), COCP (5 [4.5%]), ramipril (4 [3.6%]), salbutamol (4 [3.6%]), and atorvastatin (3 [2.7%]) most frequently reported.

**Table 1:** Table of demographics.

Characteristic	Value
Sample size	112
Age (years)	
Mean ± SD	35.3 ± 15.1
Median	31
Range	18–86
Sex	
Female	68 (60.7%)
Male	44 (39.3%)
Ethnicity	
White	69 (61.6%)
Asian	9 (8.0%)
Black/Caribbean	4 (3.6%)
Mixed	11 (9.8%)
Other	19 (17.0%)
BMI (kg/m^2^)	
Mean ± SD	24.8 ± 4.0
Median	24.4
Range	17.8–39.4
Medical conditions	
Participants with any medical condition	34 (30.6%)
Most common conditions	
Asthma	9 (8.0%)
Depression	5 (4.5%)
Hypertension	4 (3.7%)
GORD	3 (2.8%)
Current medications	
Participants on any medication	42 (37.5%)
Most common medications	
Metformin	5 (4.5%)
COCP	5 (4.5%)
Ramipril	4 (3.6%)
Salbutamol	4 (3.6%)
Atorvastatin	3 (2.7%)

### LT HR data

Heart rate data during the LT are presented in Table 2. The maximum HR value compared to baseline was used across all 10 min of standing for analysis. Mean supine HR for all participants was 71.7 ± 12.8 bpm. Upon assumption of upright posture, the mean HR change was 9.89 ± 8.15 bpm, while the mean sustained HR change (defined as maximum HR maintained over at least 2 consecutive measurements when standing) was 6.33 ± 6.82 bpm.

**Table 2:** Table breaking down LT test findings.

LT metrics	Value
Lying down supine	
Heart rate (bpm), mean ± SD	71.7 ± 12.8
Supine to standing (0–10 min)	
Average HR change (increase) (bpm), Mean ± SD	9.89 ± 8.15
Average Sustained HR change (increase) (bpm), Mean ± SD	6.33 ± 6.82

Sustained HR is defined as the maximum HR was maintained over at least 2 consecutive measurements when standing. HR value used is the maximum or minimum value compared to baseline across all 10 min of standing. Kolmogorov–Smirnov Tests of Normality were conducted, and data was found to be normally distributed.

### LT BP data

Blood pressure responses to LT are shown in Table 3. All BP measurements were normally distributed as confirmed by Kolmogorov–Smirnov tests. Both early phase standing (0–3 min) and late phase (4–10 min) standing changes were compared to supine baseline values. Mean supine systolic and diastolic BP were 120.4 ± 12.5 mmHg and 76.3 ± 9.0 mmHg, respectively. During early phase standing, the mean systolic BP increased by 7.55 ± 10.88 mmHg and mean diastolic BP increased by 4.97 ± 8.36 mmHg. In the late phase, the mean systolic BP increase decreased to 3.53 ± 10.01 mmHg and diastolic BP change by 3.31 ± 7.64 mmHg.

**Table 3:** Table breaking down LT blood pressure findings.

LT metrics	Value	
Lying down supine		
Average systolic BP (mmHg), mean ± SD	120.4 ± 12.5	
Average diastolic BP (mmHg), mean ± SD	76.3 ± 9.0	
Supine to standing	Early phase (0–3 min)	Late phase (4–10 min)
Average systolic BP change (increase) (mmHg), mean ± SD	7.55 ± 10.88	3.53 ± 10.01
Average diastolic BP change (increase) (mmHg), mean ± SD	4.97 ± 8.36	3.31 ± 7.64

Early Phase are values during the first 3 min of standing and Late Phase are values from 4 to 10 min; both changes are compared to lying down baseline value. Kolmogorov–Smirnov Tests of Normality were conducted, and data was found to be normally distributed.

### LT symptoms

Symptoms occurring across all participants during the LT are presented in Table 4. Nine participants (8.0%) reported symptoms upon assumption of upright posture. The most reported symptom was dizziness/unsteadiness (6 [5.4%]), followed by pins and needles/numbness in the legs (2 [1.8%]) and back pain (2 [1.8%]). None of these 9 participants met the critria for PoTS or OH.

**Table 4:** Table of symptoms described by participants during the LT.

Symptoms	Number of participants (%)
Participants with any symptoms on standing	9 (8.0%)
Dizziness/unsteadiness	6 (5.4%)
Pins and needles/numbness	2 (1.8%)
Back pain	2 (1.8%)

There was *n* = 4 participants who had abnormal HR/BP in their LTs. One participant had a systolic BP drop of greater than 20 mmHg, one participant had an increase of 30 bpm from lying. Two participants had persistent tachycardia above 120 bpm, these participants did not have autonomic symptoms, and tachycardia was not an exclusion criterion. All 4 participants were all asymptomatic during the LT.

We calculated changes in HR and BP, comparing gender and ethnic groups, and did not find any significant differences. HR and BP were not affected in patients taking ramipril or salbutamol.

**Figure 2:**
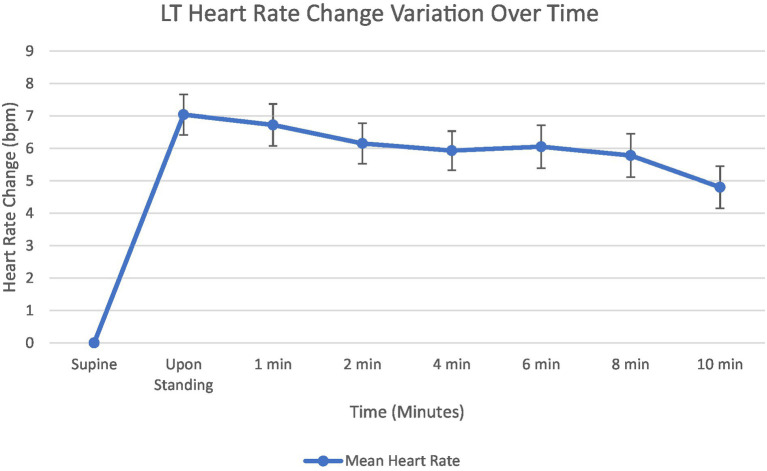
Graph showing mean heart rate change from supine at 1, 2, 4, 6, 8, and 10 min. Standard error bars.

Orthostatic increases in HR during the LT are shown in Figure 2. Mean HR increased immediately upon standing to 7.0 bpm above baseline. This initial HR increase diminished progressively over the 10-min standing period. A slight plateau was observed between 4 and 8 min with values ranging from 5.9 to 6.1 bpm above baseline. The HR increase reached its end at 4.8 bpm. The time course demonstrates an initial compensatory HR increase followed by partial recovery but with sustained elevation compared to supine values throughout the test duration.

**Figure 3:**
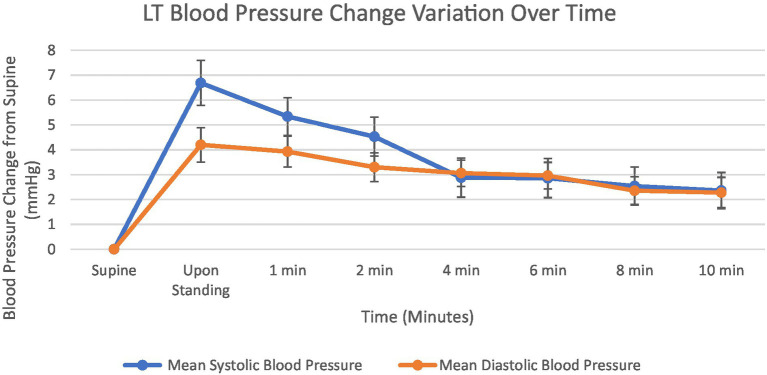
Graph showing mean systolic blood pressure and diastolic blood pressure change from supine at 1, 2, 4, 6, 8 and 10 min. Standard error bars.

Blood pressure responses to orthostatic challenge are presented in Figure 3. Both systolic and diastolic BP exhibited immediate increases upon standing, with systolic BP showing a greater magnitude of change, 6.7 mmHg, compared to diastolic BP, 4.2 mmHg. The BP increases progressively decrease over the 10-min standing period, with more pronounced declines observed in the early phase (0–3 min) than in the late phase (4–10 min). By test conclusion, systolic BP was at 2.4 mmHg above baseline, while diastolic BP showed similar values at 2.2 mmHg above baseline. The convergence of systolic BP and diastolic BP increases at approximately 4 min. Like HR, BP has an initial increase, followed by a decline and plateau, always remaining above baseline.

## Discussion

As far as we are aware, this is the first paper within academic literature to conduct LTs on a cohort without orthostatic intolerance symptoms to attain values and understanding of what is to be expected. The assumption from this is that the finding of a negative LT would help exclude OI in a patient. Normally, standing upright can lead to venous pooling subsequently reducing venous return and cardiac output, resulting in a fall in BP. Subsequently, this stimulates arterial baroreceptors with parallel activation and deactivation of sympathetic and parasympathetic nervous systems, respectively. This compensatory autonomic response restores BP within seconds through vasoconstriction and increased HR (37).

Compared with other cohorts for LTs, the largest group is the healthy population from the study by Lee et al. (34) with a population size of *n* = 50. The gender distribution was similar [64% female in the Lee et al. (34) study and 61.6% in this study]. The ethnic distribution was similar for the White category in both studies (62%), although differences were evident in the distribution of other ethnicities, particularly in the Mixed/Multiple category [28% in Lee et al. (34) study compared to 9.8% in this study]. The average BMI was nearly identical between the two cohorts [25 ± 5 kg/m^2^ in the Lee et al. (34) study and 24.8 ± 4.0 kg/m^2^ in this study]. However, there was a notable distinction in the age distribution, with Lee et al.’s (34) population being significantly older on average (48 ± 16 years) compared to this study (35.3 ± 15.1 years). The prevalence of pre-existing conditions also differed [12% in Lee et al. (34) study versus 30.6% in this study]. Hypertension was present in similar proportions [6% in Lee et al. (34) study and 3.6% in this study]. This study therefore provided a comparative normative data in a younger population that is needed while managing younger adults with OI.

The normative data from our study can be compared with LT data collected in the study by Isaac et al. (1) from 100 LC patients. The mean HR changes in that study was 18.45 ± 9.93 bpm which was significantly different from data collected in this study (unpaired *t*-test, *p* < 0.0001). This also provides further support for clinical use of LTs in screening for OI (including neurally mediated syncope). Once again, the age difference could be a confounding factor in comparison [46.6 years in Isaac et al. (1) paper vs. 35.6 years in this study] and a direct statistical significance cannot not be calculated.

Our study shows that the LT is a useful tool to detect OI in LC patients, as it is easy and safe to do either at home or in a clinic. Orthostatic Intolerance and dysautonomia can be missed in those with multiple health conditions and can cause serious problems if left untreated, hence early diagnosis and treatment are important. More research is needed to understand how OI affects LC patients over time. Health professionals who work with LC patients should learn how to assess, interpret, and manage OI and dysautonomia, and have clear guidelines for further care.

This study provides comparative data for using LT to screen for OI and dysautonomia in health conditions including LC. From this cohort of participants, we have the average HR and BP that one can expect to see in those who do not have dysautonomia or OI. The study provides some reasonable thresholds for HR and BP change beyond which one can expect to see OI symptoms. It can also be argued that the current thresholds for PoTS and OH are based on studies that used HUT test in hospital settings that are being used in LT and not necessarily validated for LT. This also explains why some LT, even though convincingly positive for symptoms, do not meet the required thresholds for PoTS or OH. Isaac et al. (1) in their study explore the alternative threshold options (for example HR change of 20 or 25 in PoTS) in LC.

The origins of the LT goes back to a paper entitled “Cardiovascular deconditioning during space flight and the use of saline as a countermeasure to orthostatic intolerance” (38) whereby the use of a tilt table test and standing test was found to be comparable, which has been further backed up by a paper by Plash et al. (39); finding that the diagnosis of PoTS using both techniques was more reliable using a 10-min standing test as the Head Up Tilt (HUT) test had lower specificity with higher false positives in healthy volunteers test. This study also suggested that the conventional thresholds used for PoTS diagnosis were based on HUT test and needed to lower when using the 10-min stand test. These studies further validate the use of LT for more widespread use in clinical practice.

The use of LT can reduce the overall cost in the clinical management for autonomic dysfunction as the LT is cheap and can be performed in home settings when compared to HUT hospital test (40). The benefit of the LT is that it uses only BP equipment which is readily available to both clinicians and patients and can be conducted in a manner that is easy to understand and replicate across locations with simple instructions. This can aid management in any health setting including primary care and community healthcare.

When comparing the normative values in a healthy population seen in this study to that of a LC population, we can see that there is significant difference in HR and BP changes in LC that correlates to symptoms during the test. This provides further validation of the presence of dysautonomia in LC and other medical conditions. The test also provides a repeatable objective test that patients and clinicians can relate to and use the test to assess response to interventions and understand condition trajectory (36). This is particularly important when there is currently no single defined biomarker identified for LC which can be used for diagnosis and monitoring.

We chose a younger population in this study to generate normative data that can be used for comparison in LC and other post-infection conditions which are common in this age group. One of the other conditions which is related to significant dysautonomia is Myalgic Encephalomyelitis/Chronic Fatigue Syndrome (ME/CFS). This condition has an early onset (41) and needs normative data to interpret LT findings. This would allow for a direct comparison and further strengthen any association that may be found between LC and ME/CFS patients with dysautonomia patients and potentially lead to clinical guidelines for objective testing and management of dysautonomia syndromes.

One of the limitations of this study is the COVID-19 status of the participants. Even though there is no history of LC or post-infection conditions, it is not known whether they had COVID-19 and fully recovered, or they truly did not have the infection during the pandemic. The infection status is however not an absolute requirement because LC and other post-infection conditions are clinical syndromes and not dependent on having confirmatory lab tests that prove the infection. It is also impractical to find individuals who have not had any infection in the past as almost everyone in the general population is subjected to infections of one type or other and most individuals make a full recovery from infections. The sample we got for this study is as best a normative cohort one can get from general population without any clinical symptoms.

It is also important to mention active stand tests (33, 42) which have a similar physiological basis to the LT used in this study. The active stand with BP/HR measurements is performed for 3-min or 6-min in total. There are two key differences which made us choose to gather normative data for the LT. The first is that the test itself, specifically the standing component of 10 min, is a longer clinical test which can provide more information on hemodynamic changes. The second is that this is a practically safer test within clinical practice as the individual undergoing testing is leaning against a surface instead of freestanding; if there is a sudden hemodynamic change, the individual has the safety of the wall to fall against compared to no aid.

In the paper by Lee et al. (32), for the healthy controls, there is a trend of increasing HR and BP upon standing from baseline followed by decreasing back toward the baseline, which is consistent with our participants. In a review on active stand tests on healthy volunteers (43) found similar findings for HR but found an inverse relationship for BP. The reasons for this are not clear yet. There is some recent concern expressed about the validity of the LT when compared to the gold standard Head Up Tilt table test (44) There is however a need for a simple test that can be used in any setting (hospital/clinic/home) and can be sensitive enough to identify those that might be having orthostatic intolerance syndromes and dysautonomia. Even though LT is not a gold standard test, it helps the general physicians in any setting to identify abnormalities and manage patients appropriately (44). Our study on healthy younger individuals provides useful normative data that will be useful when clinicians are interpreting LT findings in patients.

Our study has a few limitations. Firstly, even though all participants did not have symptoms of orthostatic intolerance, some participants had hypertension which can arguably affect the findings. However, given these participants were asymptomatic, they still provide a reliable control group for patients with orthostatic intolerance, as it is the actual fluctuation of HR and BP which determine the symptoms. Secondly, there are various versions of NLT used in the literature. We used the version used in LOCOMOTION study (34), which involved 2–5 min of lying down prior to standing. Some versions of the test involve 10–20 min of lying down (25, 32). Future research needs to explore the optimal supine time for the test that is likely to provide reliable results.

## Data Availability

The raw data supporting the conclusions of this article will be made available by the authors, without undue reservation.
